# Potential antitumoral effects of SRPK1 inhibition through modulation of VEGF splicing in pituitary somatotroph tumoral cells

**DOI:** 10.3389/fendo.2025.1667327

**Published:** 2025-10-08

**Authors:** Donatella Treppiedi, Sonia Di Bari, Federica Mangili, Anna Maria Barbieri, Genesio Di Muro, Marco Locatelli, Alessandra Mangone, Erika Peverelli, Giovanna Mantovani

**Affiliations:** ^1^ Endocrinology Unit, Fondazione IRCCS Ca’ Granda Ospedale Maggiore Policlinico, Milan, Italy; ^2^ Department of Clinical Sciences and Community Health, “Dipartimento di Eccellenza 2023-2027”, University of Milan, Milan, Italy; ^3^ Neurosurgery Unit, Fondazione IRCCS Ca’ Granda Ospedale Maggiore Policlinico, Milan, Italy; ^4^ Department of Pathophysiology and Transplantation, University of Milan, Milan, Italy

**Keywords:** SRPK1, SRSF1, VEGF, alternative splicing, pituitary tumors

## Abstract

Alternative splicing is a crucial mechanism of gene regulation that can be dysregulated in cancer. In pituitary neuroendocrine tumors (PitNETs), alteration in the serine/arginine-rich splicing factors (SRSFs) has been reported. SRSFs phosphorylation and activation is mediated by serine-arginine protein kinase 1 (SRPK1). SRPK1 is considered a proto-oncogene and its inhibition by small molecule inhibitors SRPIN340 and SPHINX31 have shown antitumoral effects via the SRPK1-SRSF1-VEGF pathway modulation in different cancer types. No previous studies have evaluated SRPK1 inhibitors in pituitary tumors. The present work explores the antitumoral effects of SRPIN340 and SPHINX31 in rat and human GH-secreting pituitary tumoral cells. First, immunoblot results showed a reduction of SRSFs phosphorylation induced by both compounds, demonstrating the efficacy of these molecules in inhibiting SRPK1 activity. SRPIN340 reduced GH4C1 cell proliferation (-31.7 (33.6)%, p <0.05 vs control cells at 1µM), cell viability (-16.4 (27.9)%, p<0.05 vs control cells at 1µM), cell migration (-65.0 (46.3)%, p<0.001 vs control cells at 10µM) and induced cell apoptosis (+40.5 (26.6)%, p<0.05 vs control cells at 10µM). Moreover, SRPIN340 significantly decreased both transcript (-56.3 (38.6)%, p<0.01 vs control cells) and protein levels (-33.5 (3.4)%, p<0.05 vs control cells) of the pro-survival VEGF164a isoform. Similar results have been obtained with SPHINX31. Interestingly, cells incubation with the recombinant VEGF164a protein impaired the decrease of cell migration and cell viability mediated by both SRPK1 inhibitors. As for GH-secreting primary cultures from GH-PitNETs, SRPIN340 incubation resulted in reduced VEGF165a expression (-50.6% vs control cells) and GH secretion (-14.45 (8.17)%, p < 0.05 vs control cells). In conclusion, SRPK1 inhibition may represent a novel approach to exert antitumoral effects in somatotroph tumoral cells via SRPK1-SRSF1-VEGF pathway regulation.

## 1 Introduction

Pituitary neuroendocrine tumors (PitNETs) comprise a heterogeneous subset of neoplasm and are divided into clinically functioning and non-functioning tumors, according to the presence or not of hormonal hypersecretion. Large scale analysis with the latest molecular biology methods that simultaneously investigate multiple factors have been employed to uncover the mechanisms of tumors development and to unveil novel biomarkers and therapeutic targets. Recently, splicing dysregulation and its subsequent outcomes has been associated to pituitary tumorigenesis and PitNETs aggressiveness (1, 2). However, the potential value of specific splicing machinery components as novel therapeutic targets in these pathologies remained to be extensively assessed.

Alternative splicing is a tightly regulated mechanism through which approximately 95% of eukaryotic genes produce multiple mRNA transcripts by the differential inclusion of specific exons and retention of intronic fragments (3). The intracellular machinery that catalyzes the whole process is the spliceosome, comprising of heterogenous nuclear ribonucleoproteins (hnRNPs), RNA-dependent ATPase/helicases and other regulatory components that cooperate with splicing factors such as the Serine/Arginine-rich splicing factors (SRSFs) (4, 5). Specifically, SRSFs belong to the Serine/Arginine-Rich (SR) proteins family characterized by one or two RNA recognition motifs (RBMs) that recognize splice sites within the mRNA, and a C-terminal SR domain enriched with arginine and serine residues involved in protein–protein interaction and subjected to several phosphorylation steps (6). In the cytoplasm, serine-arginine protein kinase 1 (SRPK1) mediates the first phosphorylation step of newly synthetized SRSFs that facilitate their nuclear import (7). Extracellular signals such as Epidermal Growth Factor (EGF) can trigger SRPK1 autophosphorylation and activation leading to SRPK1 translocation to the nucleus itself in order to accomplish a second step of SRSFs phosphorylation (8–10). Hyperphosphorylated SRSFs accumulate in nuclear speckles where the spliceosome assembly initiates and alternative splicing occurs (11, 12). SRSFs act as oncoproteins in different tumor types (13–16) and, similarly, SRPK1 altered expression plays a pivotal role in cancer (17, 18). Elevated levels of SRPK1 have been found in leukemia (19) and in several types of solid tumors, such as colon, pancreatic, lung and breast carcinomas (20–23).

The important role of splicing events in tumorigenesis can be demonstrated when looking at the control exerted by SRPK1/SRSF1 on the vascular endothelial growth factor-A (VEGF-A, hereafter referred as VEGF) gene (24). The human VEGF gene comprises eight exons and seven introns. Depending on the selection of the proximal splice-site or the distal splice-site in exon 8, two VEGF families of isoforms with opposite functions may be generated: the pro-angiogenic VEGF_xxx_a and anti-angiogenic VEGF_xxx_b families (where x denotes the number of amino acids within a given isoform, refer to 25), respectively (26, 27). SRSF1 interacts with an exonic sequence enhancer (ESE) upstream of the VEGF exon 8 proximal splice site favoring VEGF_xxx_a splicing (24, 28). Moreover, VEGF occurs in several isoforms of which the 121, 165 and 189 amino acids long forms are the most common in human (all one amino acid shorter in rodents) (29). Among these, VEGF165a (rodents VEGF164a) is the most abundantly produced during pathological angiogenesis and inflammation (30, 31). By binding to VEGF receptor 1 and 2 (VEGFR-1/2), VEGF165a activates numerous intracellular pathways resulting in promotion of angiogenesis, cell proliferation, migration and survival (29). The pituitary gland contains abundant VEGF165a as well as VEGF receptors (32–34). VEGF participates in the formation of the vascular network of the new tumor and is involved in proliferative activity of lactotrophs and somatotrophs (35, 36). Nevertheless, the use of anti-VEGF therapies such as bevacizumab and tyrosine kinase inhibitors to treat aggressive PitNETs have produced contentious results (37).

So far, several small molecules capable of inhibiting SRPK1 catalytic activity, thus reducing SRSF1 phosphorylation and proangiogenic VEGF upregulation, have been developed, such as the isonicotinamide compound SRPIN340 and its more potent derivative SPHINX31 (38–40). *In vitro* and *in vivo* studies have linked SRPIN340 and SPHINX31 antitumoral effects with the reduction of VEGF165a isoform (28, 39, 41, 42). Based on this premises, the aim of the present work is to test the *in vitro* pharmacological effects exerted by SRPIN340 and SPHINX31 in primary cultures from human GH-secreting PitNETs and in rat somatotroph tumoral GH4C1 cells, by focusing on SRPK1/SRSF1-mediated alternative splicing regulation of the VEGF gene, in the attempt to find alternative medical strategies to somatostatin receptor ligands (SRLs) for the treatment of this type of tumors.

## 2 Materials and methods

### 2.1 Pituitary cell culture

Rat pituitary tumoral GH4C1 cells (ATCC CCL-82.2) and GH3 cells (ATCC CCL-82.1) were cultured in F10 or F12K medium (Capricorn Scientific, Ebsdorfergrund, DE), respectively, supplemented with 15% horse serum (HS), 2.5% fetal bovine serum (FBS), 2 mM glutamine and antibiotics, all supplied by Gibco (ThermoFisher Scientific, Waltham, MA, USA). Primary cell cultures from four different GH-secreting PitNETs were obtained from surgically removed human tumors, as previously described (43). Cells were maintained in DMEM medium (Gibco, Invitrogen, Life Technologies Inc., Carlsbad, CA, USA) supplemented with 10% FBS and 2 mM of glutamine and antibiotics. This study (#1167_2022) was approved by the local Ethics Committee (Comitato Etico Milano Area 2), and each patient gave informed consent.

### 2.2 Chemicals

SPRIN340 and SPHINX31 were from TargetMol (TargetMol Chemicals Inc, Boston, MA, USA). Powders were dissolved in sterile DMSO (Sigma-Aldrich; St. Louis, MO, USA) at the 50 mM concentration, stored at -80°C and diluted in PBS immediately before use. EGF was purchased from Peprotech (Thermo Fisher Scientific, Waltham, MA, USA), reconstitute in water and used at 100 ng/ml concentration. Recombinant Rat VEGF164a (Bio-techne, Minneapolis, MN USA) was resuspended in PBS with 0,1% BSA (Sigma-Aldrich; St. Louis, MO, USA) and used at 50 ng/ml concentration.

### 2.3 Immunofluorescence

For SRPK1 subcellular localization analysis, GH4C1 cells were seeded on 13-mm poly-L-lysine coated coverslips at a density of 1.25 × 10^5^ cells/well in 24-well plates and let in starved medium at 37°C for 18 h. The following day cells were incubated with SRPIN340 or SPHINX31 (10 µM) for 2 h, then EGF (100 ng/ml) was added for 1 h. At the end of the incubation, cells were chilled on ice and fixed with 4% paraformaldehyde (Sigma-Aldrich, St. Louis, MO) for 10 min at room temperature. After washing with PBS three times, cells were blocked with blocking buffer for 1 h. Then coverslips were loaded with primary SRPK1 antibody (1:100, BD Biosciences, Franklin Lakes, NJ, USA), incubated o/n at 4°C, washed with PBS three times, and treated with anti-mouse Alexa Fluor™−488-conjugated secondary antibodies (1:500, ThermoFisher Scientific, CA) for 2 h at room temperature. Both primary and secondary antibodies were diluted in Antibody Diluent Reagent Solution (Invitrogen, ThermoFisher Scientific, Waltham, MA, USA). Washed coverslips were mounted with ProLong^®^ Diamond antifade mountant containing 4,6-diamidino-2-phenylindole (DAPI) (Biotium, Fremont, CA, USA). Images were captured on a fluorescence microscope (Zeiss Axio Vert.A1, Carl Zeiss, Oberkochen, DE). Experiments were repeated three times.

### 2.4 Western blot analysis

For analysis of serine-arginine protein phosphorylation (pSR) status of the SRSFs, GH4C1 cells were o/n seeded at a density of 5 × 10^5^ cells/well in 6-well plate in starved medium, then incubated with 10 µM of SRPIN340 or SPHINX31 for 2 h, and further treated with EGF (100 ng/ml) for 1 h. Cells were lysed with lysis buffer (Cell Signaling Technology, Danvers, MA, USA) and total proteins were quantified by bicinchoninic acid assay (BCA). 60 µg proteins were separated on SDS/polyacrylamide gels and transferred to a nitrocellulose filter. pSR primary antibody mAb1H4 from Sigma-Aldrich (Sigma-Aldrich, St. Louis, MO, USA) is able to detect different phospho-SR proteins epitopes and was incubated o/n at 4°C (44). Experiments were repeated at least three times. To evaluate SRPK1 expression in GH4C1 cells and GH3 cells and check SRPK1 silencing efficiency in GH4C1 cells, 60 µg of total protein were separated on SDS/polyacrylamide gels and transferred to a nitrocellulose filter. An anti-SRPK1 antibody from BD Biosciences (Franklin Lakes, NJ, USA) was diluted 1:1000 and incubated o/n at 4°C. To determine the expression levels of VEGF165a in human primary cultured cells from one GH-secreting PitNET (#2), cells were exposed or not to SRPIN340–100 nM for 4 h, then cells were lysed and 20 µg of total proteins were loaded on SDS/polyacrylamide gel. A specific antibody was selected from Bio-techne (Minneapolis, MN, USA, clone #26503, R&D Systems) and incubated o/n at 4°C at 1 µg/ml dilution. For normalization, all membranes were incubated with anti-GAPDH antibody (Invitrogen, ThermoFisher Scientific, Waltham, MA, USA) diluted 1:4000, 1 h at room temperature. Secondary antibodies anti-mouse (Cell Signaling, Danvers, MA, USA) were used at 1:2000 at room temperature for 1 h. Chemiluminescence was detected using UVP ChemiDoc-it™ Imaging System (UVP, Upland, CA, USA) and bands were subjected to densitometrical analysis by the use of NIH ImageJ software.

### 2.5 SRPK1 genetic silencing

Small interfering RNAs (siRNAs) against rat SRPK1 gene were purchased from Dharmacon (smart pool siRNA, GE Healthcare Life Sciences, Chicago, IL, USA). Dharmafect transfection agent 2 (Dharmacon, GE Healthcare Life Sciences, Chicago, IL, USA) was used. GH4C1 cells were transiently transfected with siRNAs following the instructions of the manufacturer for 72 h. In each experiment a negative control siRNA (non-targeting sequence without significant homology to the sequence of human, mouse or rat transcripts) was used. SRPK1 silencing efficiency was tested by Western blot and only experiments achieving at least 70% silencing were considered.

### 2.6 Cell proliferation assay

5-Bromo-2’-deoxyuridine (BrdU) incorporation during DNA synthesis in proliferating GH4C1 cells was measured with specific ELISA kit (Roche, Basilea, Switzerland). Briefly, cells were o/n seeded in starved medium in 96-well poly-lysine-coated plate at a density of 4 × 10^4^ cells/well. The following day, starved medium was replaced with fresh complete medium containing SPRIN340 or SPHINX31 (at different doses) for 72 h. BrdU incorporation in newly synthesized DNA was then allowed at 37°C for 2 h and the assay was performed in accordance with the instruction of the manufacturer. Each determination was performed in triplicate and experiments were repeated at least five times.

### 2.7 Cell viability

The MTT colorimetric assay, that correlates the number of metabolically active cells to the mitochondrial enzymes capability to convert MTT (3-(4,5-dimethylthiazol-2-yl)-2,5-diphenyltetrazolium bromide, Sigma-Aldrich, St. Louis, MO, USA) into formazan, was used to test cell viability of GH4C1 cells. Cells were seeded at a cell density of 1.5 × 10^4^ cells/well in 96-well plate in complete medium. The day after, cells were exposed to increasing concentration of SRPIN340 and SPHINX31 for 72 h and to 50 ng/ml of rat VEGF164a (when indicated). Medium was then replaced with DMEM without phenol red and a final concentration 5 mg/ml of MTT was added to the cells. A further incubation at 37°C for 1 h allowed to the formation of purple-colored formazan crystals, subsequently solubilized by DMSO (100 µl for each well). A Victor Nivo multimode plate reader (Perkin Elmer, Whaltam, MA, USA) was used to read absorbance at a wavelength of 560 nm. Each determination was done in triplicate and experiments were repeated at least three times.

### 2.8 Cell apoptosis assay

The enzymatic activity of caspase-3/7 was measured in GH4C1 cells with the ApoONE Homogenous Caspase-3/7 Assay (Promega, Madison, WI, USA). Cells were seeded in complete medium at the cell density of 2 × 10^4^ cells/well. 24 h after seeding, cells were incubated with SRPIN340 or SPHINX31 (1 or 10 µM) for 48 h. Following SRPK1 inhibitors incubation, a profluorescent caspase-3/7 consensus substrate diluted in a specific lysis buffer was added. A Victor Nivo multimode plate reader (Perkin Elmer, Whaltam, MA, USA) was used to read rhodamine 110 fluorescence, released upon cleavage on the C-terminal side of its aspartate residue by caspase-3/7 enzymes. The amount of fluorescent product generated was representative of the amount of active caspase-3/7 present in the sample. Each measurement was done in triplicate and experiments were repeated at least three times.

### 2.9 Transwell migration assay

Cell migration has been tested by transwell assays. GH4C1 cells were previously serum deprived for 24 h. Then, a suspension of 3 × 10^5^ cells/insert was plated in 300 μl of serum-free medium in 24-well inserts with polycarbonate membrane of 8 μm pore diameter (Merck Millipore, Darmstadt, Germany). 400 μl of complete medium was added in the lower chamber, with FBS and HS working as chemo-attractants. Cells were allowed to migrate for 18 h at 37°C with the indicated treatments (SRPIN340 1 μM or 10 μM, SPHINX31 1 μM or 10 μM, rat VEGF164a 50 ng/ml). Cells that did not migrate to the lower compartment were mechanically removed with a cotton swab, whilst migrated cells were stained for 10 min in crystal violet solution (0.5% crystal violet in 20% methanol/water). 10% acetic acid was used to extract stained cells and absorbance at a wavelength of 560 nm was recorded by a Victor Nivo multimode plate reader (Perkin Elmer, Whaltam, MA, USA). A negative control with serum-free medium in the lower chamber was used in each experiment and subtracted. For each experiment measurements were done in triplicate and experiments were replicated at least three times.

### 2.10 RT-PCR and quantitative real-time RT-PCR

Total RNA was extracted from GH4C1 with RNeasy Plus mini kit (Qiagen, Hilden, Deutschland), according to the manufacturer’s instructions. Total RNA concentration and purity were determined using a NanoDrop Lite Spectrophotometer (ThermoFisher Scientific, Waltham, MA, USA). RNA integrity was assessed by 1% agarose gel electrophoresis. 1 µg of total RNA was reverse transcribed with a RevertAid H Minus First Strand cDNA Synthesis Kit (ThermoFisher Scientific, Waltham, MA, USA). cDNA was used for qRT-PCR performed with SsoFast™ EvaGreen^®^ Supermix (Bio-Rad Laboratories, Hercules, CA, USA) following the manufacturer’s instructions, in a QuantStudio™ 3 Real-Time PCR System (Applied Biosystems, Thermo Fisher Scientific, Waltham, MA, USA). Specific primers were designed for rat *srpk1* and *srsf1* (*srpk1* forward: 5’-TTACACGCGAGATGAAGACC-3’, reverse: 5’-CTTGGTGATGTGTTTCAGGT-3’; *srsf1* forward: 5’- ATGAGGGAGAAACTGCCTAC-3’, reverse: 5’- CTACGGCTTCTGCTACGACT-3’). To carry out quantitative Real time RT-PCR of rat *vegf164a* and *vegf165b*, isoforms, GH4C1 cells were pre-treated with the indicated stimuli for 3 h and total RNA was extracted from about 1 x 10^6^ cells per condition. Specific primers were designed (*vegf164a* forward: 5′-CCAGAAAATCACTGTGAGCCTTG-3′, reverse: 5′-GGCTTGTCACATCTGCAAGTAC-3′; *vegf165b*, forward 5′-CCAGAAAATCACTGTGAGCCTTG-3′; reverse: 5′-GGTGAGAGGTCTGCAAGTAC-3’). Target gene expression was normalized to the expression of the housekeeping gene GAPDH (*gapdh* forward: 5’-GTCTCCTGTGACTTCAACAG-3’, reverse 5’-CATTGTCATACCAGGAAATGAG-3’) using the QuantStudio™ Design & Analysis Software. The relative quantification of target genes was calculated using the ΔCt method. For each experiment measurements were done in triplicate and experiments were repeated at least three times.

### 2.11 Measurement of VEGF levels

An ELISA immunoassay kit from Bio-techne (Minneapolis, MN, USA) was used to measure rat VEGF164a levels in the culture media of GH4C1 cells. Cells were seeded in 24-well plate, at a density of 1,5 × 10^5^ cells/well, in 300 μl of starved medium according to previous reported protocols (Lohrer et al., 2001). The day after, cells were stimulated with 10µM SRPIN340 or SPHINX31 for 24 h. After treatment, culture media were collected and centrifugated at 1000g for 20 min at room temperature before the assay. Absorbance was read at 450 nm in a Victor Nivo multimode plate reader (Perkin Elmer, Whaltam, MA, USA). Data were plotted and analysed with the specific Curve Expert 1.4 program. VEGF164a detection were performed in triplicate and experiments were repeated at least three times.

### 2.12 Measurements of GH levels

Primary cells from 4 different GH-secreting PitNETs (#1, #2, #3, #4) were seeded at the cell density of 4 × 10^4^ cells/well in p96well plate in 100 µl of complete media. The day after, culture media was replaced with 100 µl of fresh media containing SRPIN340 100nM for 4 h, then collected and GH was measured by specific chemiluminescent immunometric assay (Immulite 2000, Siemens Medical Solutions Diagnostics, Los Angeles, CA, USA) with an analytical sensitivity of 0.01 ng/ml. Specifically, measurements were done in quintuplicate for primary cultures #1, #2 and #4, and in triplicate for primary culture #3. Wells with fresh media without SRPIN340/Octreotide was used as control for each primary culture.

### 2.13 Statistical analysis

Variables were reported as median and interquartile range (IQR) along the text and in all graphs. To assess the significance between two series of data the non-parametric U of Mann-Whitney or Wilcoxon tests, for independent or dependent data, respectively, were applied. Moreover, significance between more than two groups of data has been evaluated with the non-parametric Friedman or Kruskal Wallis one-way ANOVA test without Dunn’s *post hoc* test correction. Data were analyzed with GraphPad Prism 10.0 software (GraphPad Software, Inc., La Jolla, CA). p < 0.05 was accepted as statistically significant.

## 3 Results

### 3.1 SRPIN340 and SPHINX31 prevent SRPK1 nuclear translocation and activity in GH4C1 cells

**Figure 1:**
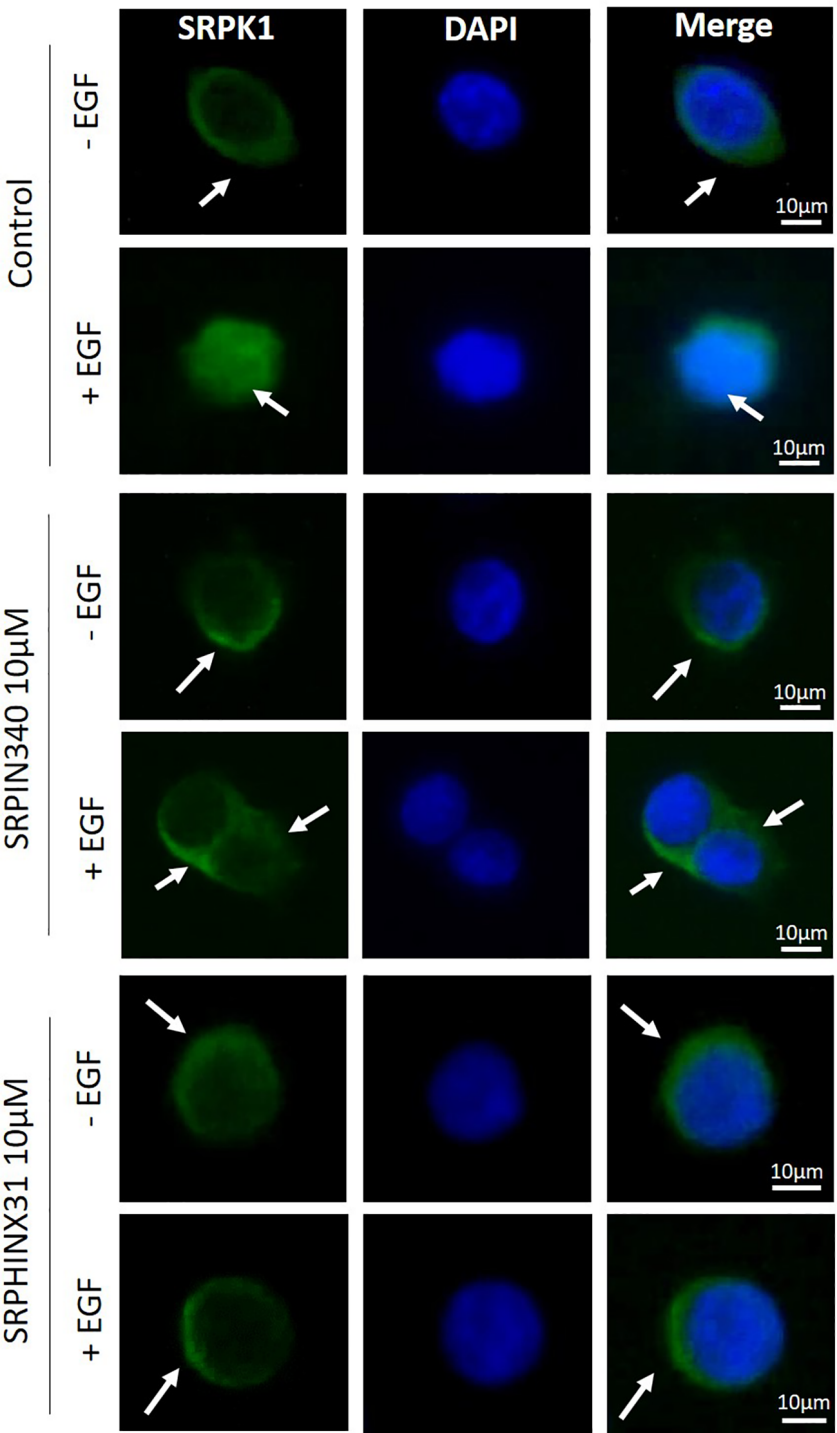
Effect of SRPIN340 and SPHINX31 on SRPK1 localization. Representative immunofluorescence images of SRPK1 in GH4C1 cells exposed to SRPIN340 or SPHINX31 (10µM) or not for 2 h and treated with EGF for 1 h. SRPK1 is represented in green whereas nuclei were counterstained in blue with DAPI. White arrows are shown to indicate subcellular localization of SRPK1 (cytoplasmatic or nuclear). The images shown are representative of three independent experiments. Scale bar: 10µm.

First, GH4C1 cells and GH3 cells were tested for SRPK1 expression. Western blot analysis showed that GH4C1 cells expressed higher level of SRPK1 compared to GH3 cells (data not shown). This data prompted us to continue exploring SRPK1 inhibitors effects in GH4C1 cells. SRPIN340 and SPHINX31 were selected as SRPK1 inhibitors that irreversibly target the kinase ATP-binding clefts. Immunofluorescence experiments were carried out to evaluate the impact of SRPIN340 and SPHINX31 on the intracellular localization of SRPK1. GH4C1 cells were incubated for 2 h with 10µM of SRPIN340 or SPHINX31 followed by 1 h stimulation with EGF 100 ng/ml. Indeed, EGF stimulation has been previously shown to be able to trigger SRPK1 autophosphorylation and activation in the nuclei (12). Under basal condition, our imaging data showed a predominant staining of SRPK1 in the cytoplasm, whereas stimulation with EGF promoted a robust SRPK1 translocation to the nucleus (**Figure 1**, upper panel). However, in the presence of SRPK1 inhibitors, SRPK1 remained predominantly localized in the cytoplasm even after cells exposure to EGF (**Figure 1**, middle and lower panel), indicating a defective SRPK1 kinase activity over itself.

**Figure 2:**
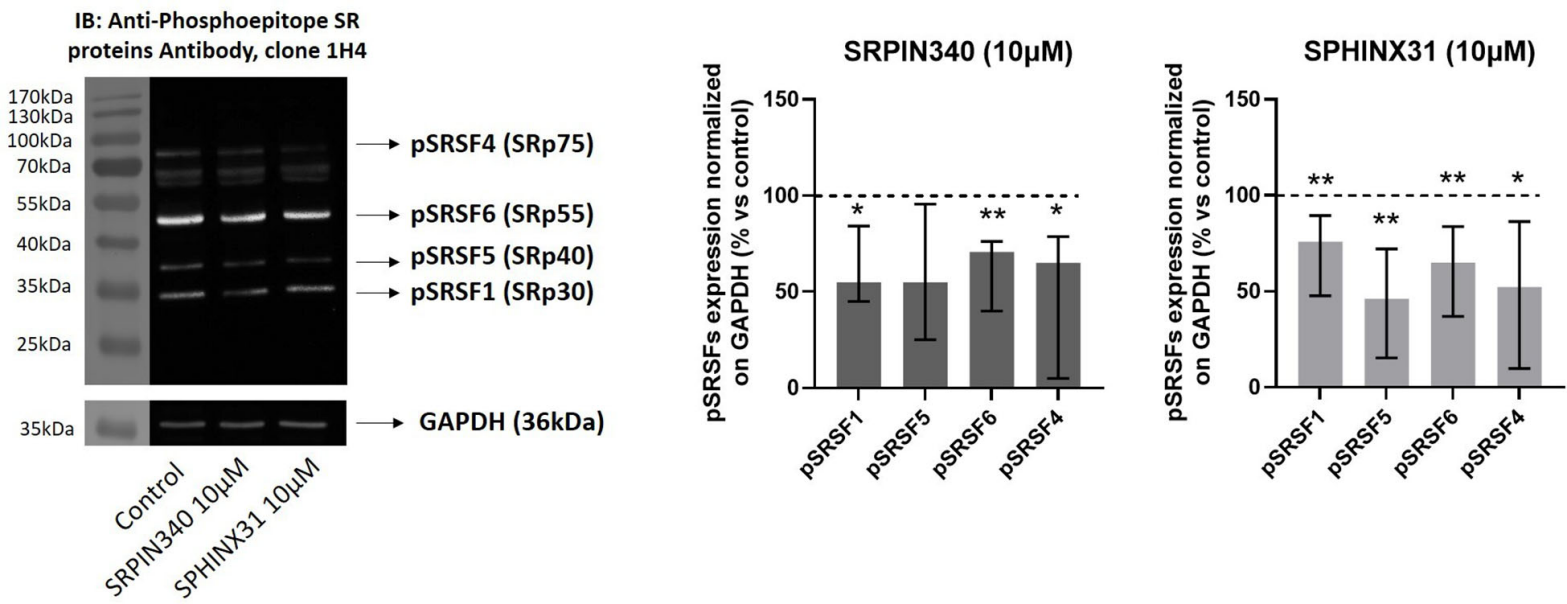
Effect of SRPIN340 and SPHINX31 on SRPK1 activity. Representative Western blot images and densitometrical analysis of phosphorylated SR proteins (pSRSF1, pSRSF5, pSRSF6, pSRSF4) related to their respective GAPDH loading control from GH4C1 cells treated with SRPIN340 or SPHINX31 (10µM) for 2 h and with EGF for 1 h. Cells treated with EGF only were used as control. Experiments were repeated at least three times. *p < 0.05 and **p < 0.01 vs control.

We further investigated the effect of SRPIN340 and SPHINX31 on SRPK1 intracellular activity by evaluating the phosphorylation level of SRSFs with an anti-phosphoepitope SR proteins antibody in Western blot experiments (44). As shown in **Figure 2**, a significant reduction of phospho-SRSF1 (-45.1 (39.2) %, p < 0.01), phospho-SRSF6 (-29.3 (36.2) %, p < 0.01) and phospho-SRSF4 (-34.9 (73.8) %, p < 0.05) was observed in SRPIN340-treated cells compared with control cells (EGF-treated cells, only). Similarly, SPHINX31 reduced phospho-SRSF1 (-24.2 (45.2) %, p < 0.05), phospho-SRSF5 (-54.0 (56.7) %, p < 0.01 phospho-SRSF6 (-35.0 (46.8) %, p < 0.01) and phospho-SRSF4 (-47.9 (76.5) %, p < 0.05) compared with control cells (EGF-treated cells, only).

### 3.2 SRPIN340 and SPHINX31 exert cytostatic and cytotoxic effects in GH4C1 cells

**Figure 3:**
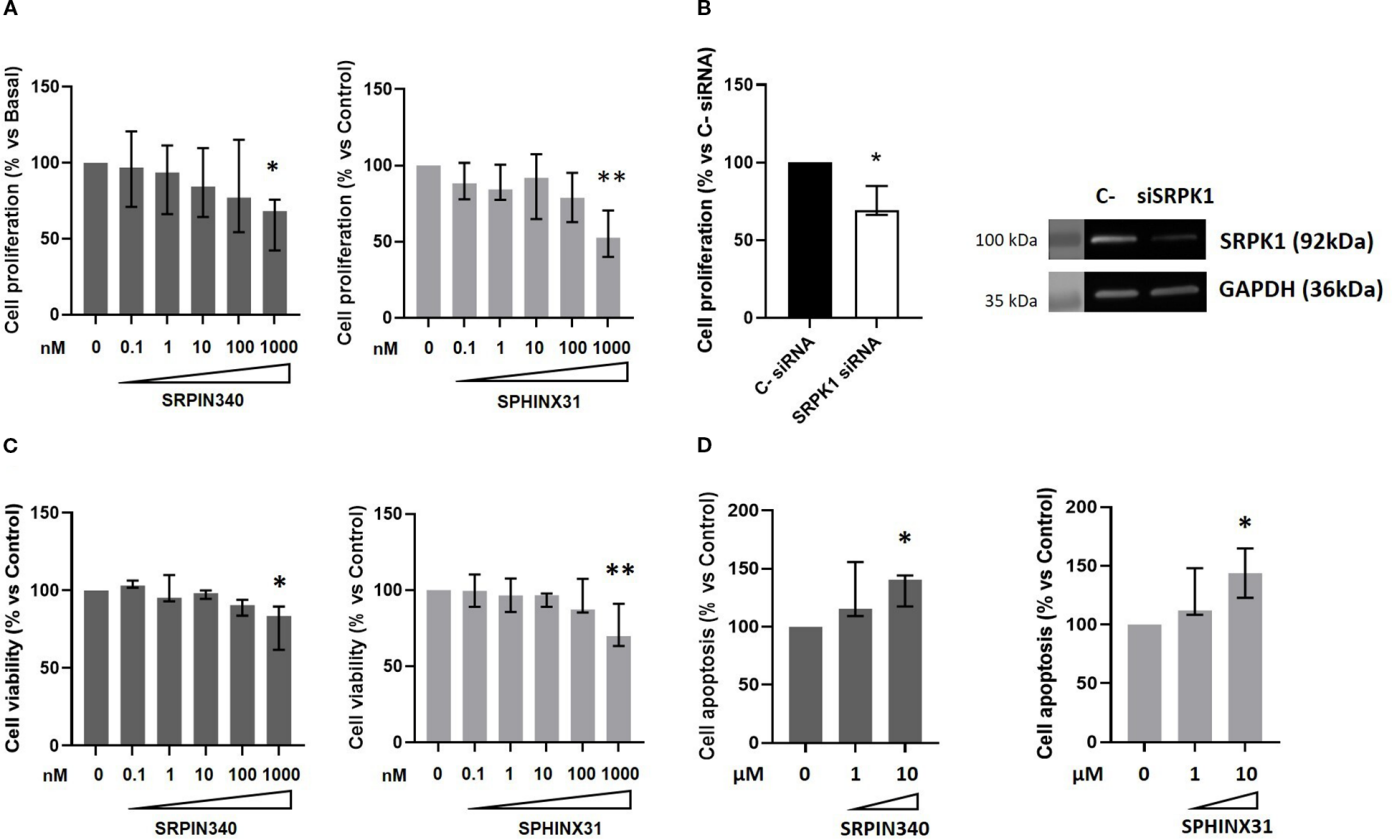
SRPIN340 and SPHINX31 action on cell growth and cell apoptosis. Increasing concentration (0.1 nM – 1000 nM) of SRPIN340 or SPHINX31 were tested in GH4C1 cells in cell proliferation **(A)** and cell viability **(C)** experiments for 72h, by BrdU incorporation assay and MTT assay, respectively. **(B)** Cell proliferation in GH4C1 cells silenced for SRPK1 for 72 and corresponding representative immunoblotting indicating SRPK1 silencing are shown. **(D)** Cell apoptosis measured by caspase3/7 activity in GH4C1 cells treated with two doses of SRPIN340 or SPHINX31 (1µM-10µM) for 48 h For each set of analysis, each determination was done in triplicate and experiments were repeated at least three times. *, p < 0.05 and **, p < 0.01 *vs* control untreated cells or control siRNA transfected cells.

We then performed dose-response experiments with SRPIN340 and SPHINX31 to test whether they could inhibit cell growth and induce cell apoptosis in GH4C1 cells. A statistically significant reduction of cell proliferation was observed at the dose of 1 µM for both compounds (-31.7 (33.6) %, p < 0.05 vs control untreated cells for SRPIN340-treated cells; -47.5 (30.5) %, p < 0.01 vs control untreated cells for SPHINX31-treated cells) (**Figure 3A**). Furthermore, SRPK1 knock down in GH4C1 cells resulted in cell proliferation reduction (-30.8 (18.5) %, p < 0.05 vs C- siRNA) (**Figure 3B**). Then, cell viability was tested. SRPIN340 and SPHINX31 slightly but significantly reduced cell viability in GH4C1 cells at 1 µM concentration (-16.4 (27.9) %, p < 0.05 vs control untreated cells for SRPIN340-treated cells and -29.9 (27.6) %, p < 0.01 vs control untreated cells for SPHINX31-treated cells) (**Figure 3C**). Two doses of SRPK1 inhibitors were tested in cell apoptosis experiments (1 µM and 10 µM). Only the 10 µM dose of either SRPIN340 or SPHNX31 was able to induce cell apoptosis in GH4C1 cells (+40.5 (26.6) %, p < 0.05 vs control untreated cells and +43.9 (42.0) %, p < 0.05 vs control untreated cells, for SRPIN340 and SPHINX31-treated cells, respectively) (**Figure 3D**).

### 3.3 SRPK1 inhibition reduces cell migration in GH4C1 cells

**Figure 4:**
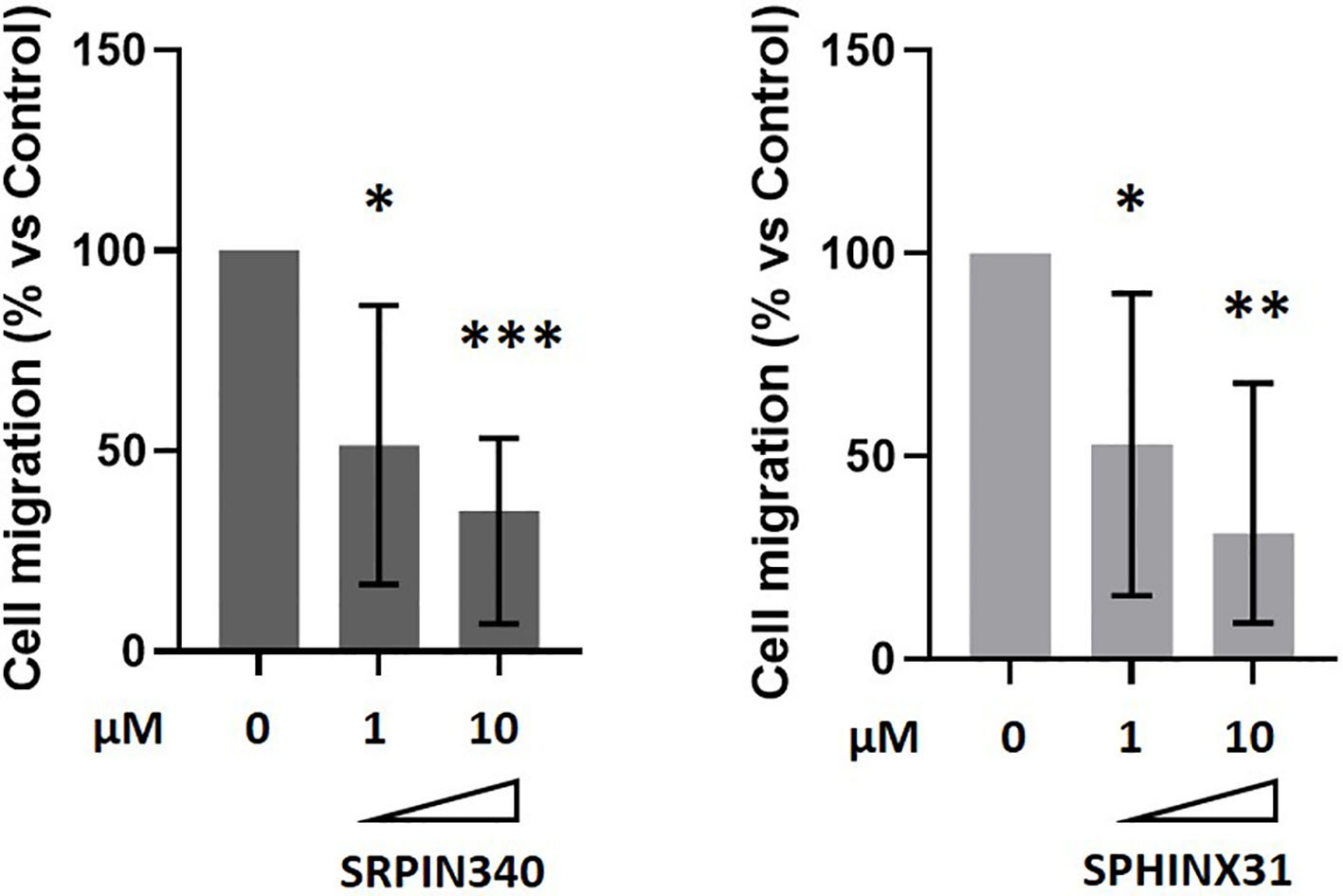
Anti-migratory effects of SRPIN340 and SPHINX31. Analysis of boyden chamber assay showing the quantification of GH4C1 cells migrated on the bottom part of the membrane after 18 h exposure to 1µM or 10µM of SRPIN340 or SPHINX31. Each determination was done in triplicate and experiments were repeated at least three times. *p < 0.05, **p < 0.01 and ***p < 0.001 vs control untreated cells.

We further verified whether SRPK1 inhibition could have an impact on GH4C1 cells migration by means of transwell migration assays and testing two different doses of SRPK1 inhibitors (1 µM and 10 µM). Either SRPIN340 or SPHINX31 exerted a strong anti-migratory action in GH4C1 cells, both achieving a maximum effect at the 10 µM dose (-65.0 (46.3) %, p<0.001 vs control untreated cells in SRPIN340-treated cells; -69.0 (59.2) %, p < 0.01 vs control untreated cells in SPHINX31-treated cells) (**Figure 4**).

### 3.4 SRPIN340 and SPHINX31 reduce the pro-angiogenic VEGF164/165a isoform levels

**Figure 5:**
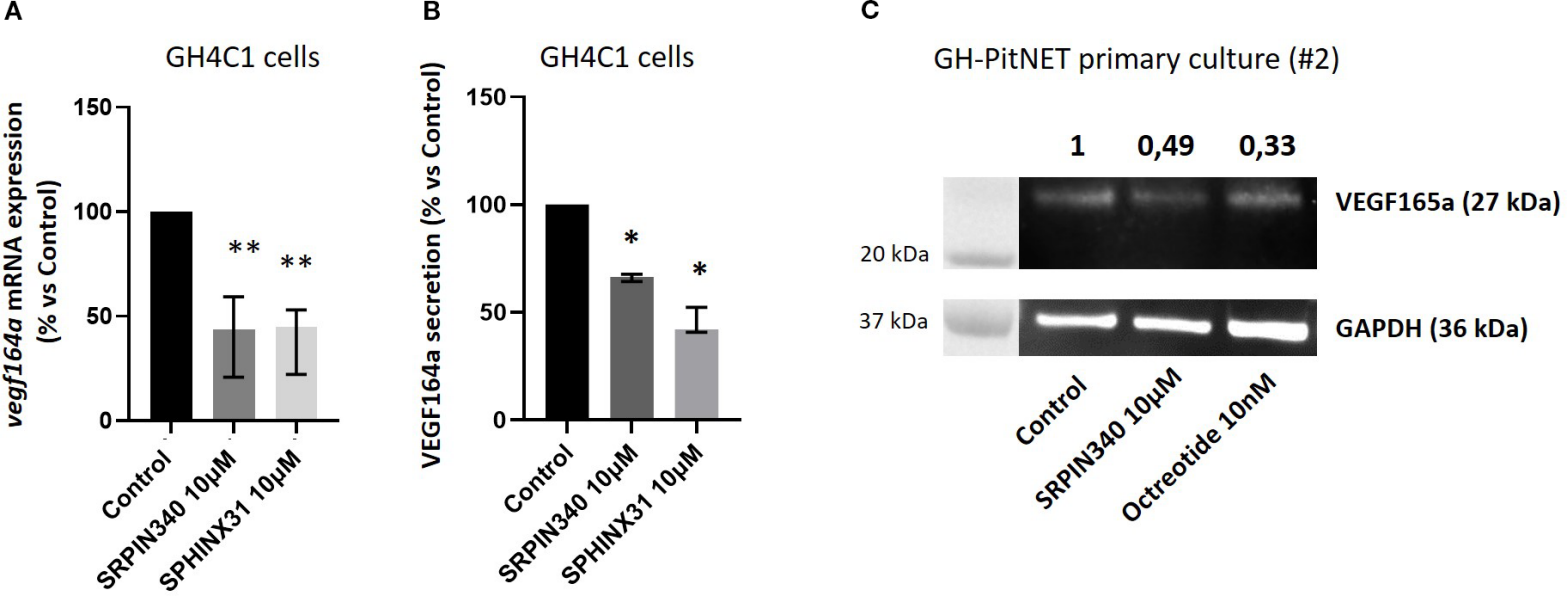
Effects of SRPIN340 and SPHINX31 on VEGF164/165a expression. **(A)** Analysis of *vegf164a* mRNA expression by RT-qPCR in GH4C1 cells treated with SRPIN340 or SPHINX31 (10 µM) for 3 h Untreated cells were used as control. Each determination was done in triplicate and experiments were repeated at least three times. **p<0.01 vs respective control. **(B)** VEGF164a protein levels were measured by ELISA immunoassay in culture media of GH4C1 cells incubated with SRPIN340 or SPHINX31 (10 µM) for 24 h Each determination was done in triplicate and experiments were repeated at least three times. *p < 0.05 vs control untreated cells. **(C)** Immunoblotting showing VEGF165a protein expression in one GH-PitNET primary culture (2#). Cells were stimulated with SRPIN340 100 nM or octreotide 10 nM for 3 h Densitometrical analysis refers to VEGF165a/GAPDH ratio and values are related to control untreated cells.

In order to unveil the molecular mechanisms responsible for the biological effects exerted by SRPK1 inhibitors in GH4C1 cells, we looked at the splicing pattern of transcripts encoding for VEGF, being this influenced by SRSF1 and SRPK1 activity. Our RT-qPCR data showed that cells incubation with SRPIN340 or SPHINX31 induced a significant transcript reduction of the pro-angiogenic and pro-mitotic *vegf164a* isoform (-56.3 (38.6) %, p < 0.01 and -55.0 (30.9) %, p < 0.01 vs control untreated cells, in SRPIN340- and SPHINX31-treated cells, respectively), (**Figure 5A**). The anti-angiogenic and anti-mitotic *vegf165b*, isoform was almost undetectable due to extremely low expression level (**Supplementary Figure 1**). Accordingly, the release of VEGF164a isoform in the culture media was significantly reduced after cells exposure to SRPIN340 (-33.5 (3.4) %, p < 0.05 vs control untreated cells) and SPHINX31 (-57.9 (11.6) %, p < 0.05 vs control untreated cells) (**Figure 5B**). In agreement, exposure to SRPIN340 reduced VEGF165a protein expression levels in one primary culture obtained from a GH-secreting PitNET (-50.6% vs control untreated cells) and, similarly, octreotide exerted the same effect (-66.4% vs control untreated cells) (**Figure 5C**).

### 3.5 The recombinant VEGF164a protein contrasts the antitumoral effects mediated by SRPK1 inhibitors

**Figure 6:**
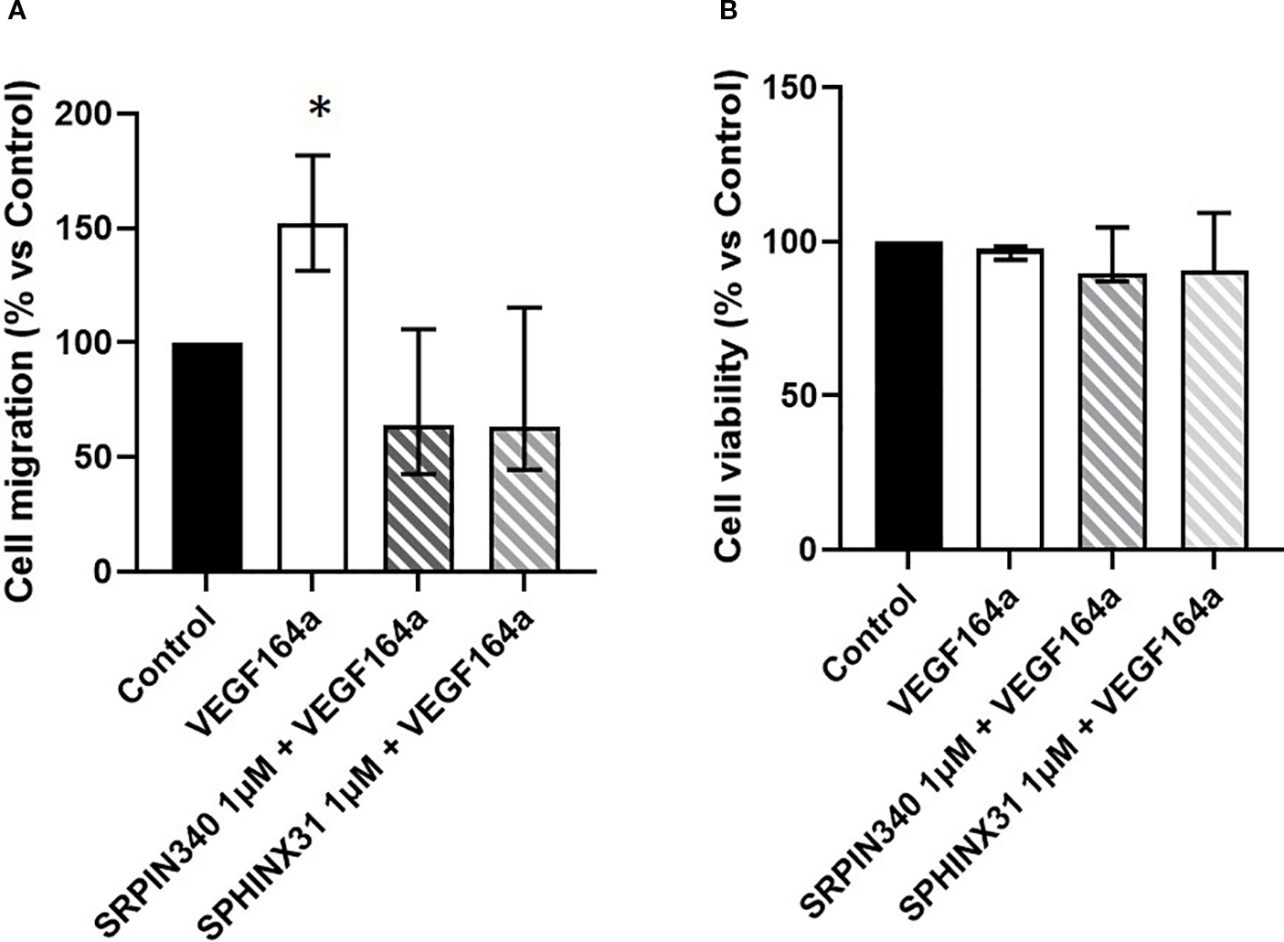
Antitumoral effects of SRPK1 inhibitors are reverted by recombinant VEGF164a. **(A)** Analysis of cell migration and **(B)** cell viability tested in GH4C1 cells co-incubated with the recombinant rat VEGF164a protein (50 ng/ml) and SRPIN340 or SPHINX31 (1 µM) for 18 and 72 h, respectively. Each determination was done in triplicate and experiments were repeated at least three times, *p < 0.05 vs respective untreated control.

To further test the hypothesis of SRPIN340 and SPHINX31 playing antitumoral effects through modulation of VEGF splicing, cell migration and cell viability were analysed in GH4C1 cells co-incubated with the recombinant VEGF164a protein and SRPK1 inhibitors. Indeed, the recombinant VEGF164a protein binds to VEGF receptor 1 (VEGFR-1) which is abundantly expressed in GH4C1 cells (data not shown). Based on preliminary experiments, the 50 ng/ml dose of VEGF164a was chosen. An induction of cell migration was observed upon stimulation with VEGF164a. Interestingly, the ability of SRPIN340 and SPHINX31 to reduce cell migration was impaired when cells were co-incubated with VEGF164a (**Figure 6A**). In addition, though VEGF164a had no effect on cell viability, it completely abrogated the capability of SRPK1 inhibitors to exert cytotoxic actions (**Figure 6B**).

### 3.5 GH secretion is diminished by SRPIN340 in GH-secreting PitNETs primary cultured cells

**Figure 7:**
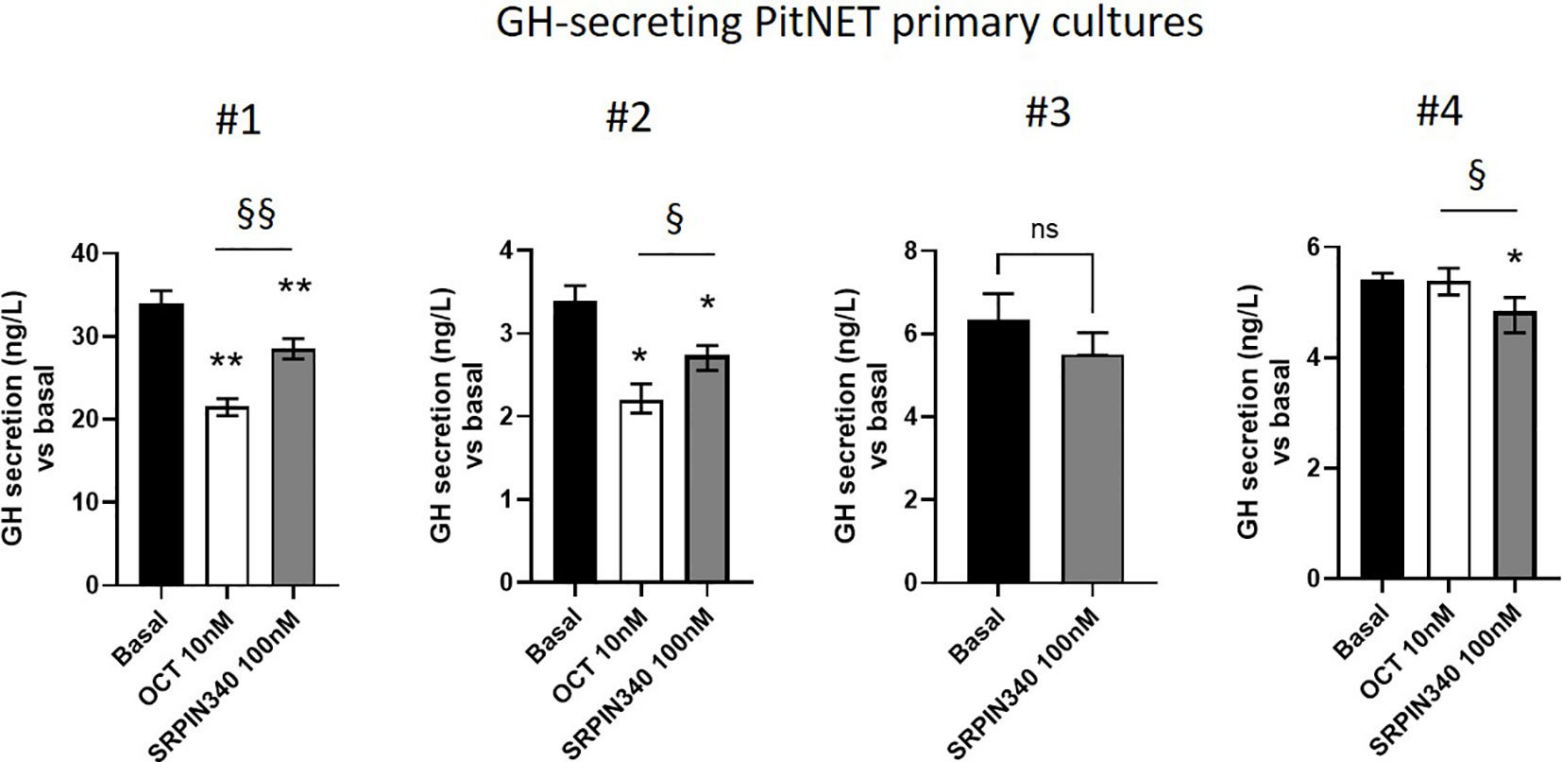
GH secretion is diminished by SRPIN340. Four different primary cultures were stimulated for 4 h with SRPIN340 100 nM or octreotide 10 nM as indicated, and GH released in the culture media as measured with specific assay. Variables were reported as median and interquartile range (IQR) of technical replicates. Specifically, measurements were done in quintuplicate for primary cultures #1, #2 and #4, and in triplicate for primary culture #3. *p<0.05 SRPIN340 vs control untreated cells, §, p<0.05 and §§, p<0.01 SRPIN340 vs octreotide-treated cells.

We finally investigated whether the SRPK1-SRSF1 pathway could modulate GH secretion in primary cultures from 4 different GH-secreting PitNETs whose clinical and pathological features are summarized in supplementary table 1 (**Supplementary Table 1**). So far, SRPIN340 and SPHINX31 showed similar effects in all experiments conducted in GH4C1 cells, thus SRPIN340 was selected as representative SRPK1 inhibitor. Dose-response experiment carried out in one primary culture allowed us to set the 100 nM dose as the lowest one able to show antisecretory effects. Considering all GH-secreting primary cultures tested, incubation with SRPIN340 resulted in a slight but significant GH secretion reduction with a median GH reduction of -14.45 (8.17)% compared with control untreated cells. Specifically, two primary cultures significantly responded to both SRPIN340 and octreotide (#1 and #2), although octreotide was more effective in reducing GH levels, whereas one primary culture that resulted *in vitro* resistant to octreotide still showed responsiveness to SRPIN340 treatment (#4) (**Figure 7**).

## 4 Discussion

The current study is the first to report anti-tumoral effects of small molecule SRPK1 inhibitors in pituitary tumoral somatotroph cells, via regulation of alternative splicing of the VEGF gene. Firstly, our imaging data and Western blot analysis in GH4C1 cells demonstrated that both SRPK1 inhibitors SRPIN340 and SPHINX31 are able to prevent EGF-induced SRPK1 nuclear translocation and impair SRPK1 ability to phosphorylate several SRSFs, including its main studied target SRSF1, thus confirming the blockade of SRPK1 activity. According to the literature, the balance between cytoplasmic and nuclear SRPK1 levels is critical for the cell. In general, a growth factor/hormone-mediated increase in the nuclear concentration of SRPK1 alters the level of phosphorylation of SRSFs and consequently their activity on their primary transcript targets, favouring the expression of splicing isoforms that contribute to cell proliferation and promote tumorigenic properties (12). In the present study, we observed an inhibitory effect on cell proliferation and cell viability mediated by both SRPIN340 and SPHINX31. SRPK1 knock down reduced cell proliferation, as well. Similarly, targeting SRPK1 in different cell lines and in *in vivo* studies resulted as a promising strategy to obtain anti-tumoral effects. Indeed, SRPK1 knockdown carcinoma LS174t cells implanted subcutaneously in a xenograft colorectal tumour model grew significantly slower compared with cells expressing a lentiviral control (28).

VEGF is a key molecule involved in angiogenesis and cell survival. Alternative splicing of VEGF gene is under tight control of SRPK1-mediated SRSF1 phosphorylation which favours proximal splicing site usage in VEGF gene, and results in augmented expression of the pro-survival VEGF165a isoform (referred as VEGF164a in rodents) (24, 45). We demonstrated that GH4C1 cells exposure to SRPIN340 or SPHINX31 results in VEGF164a transcript and protein levels reduction. However, due to extremely low mRNA levels, we were unable to consistently measure the expression of the corresponding anti-mitotic and anti-angiogenic isoform harbouring from a distal splicing site usage in the VEGF gene, neither after prolonged SRPK1 inhibition. Contrary to other studies in which knockdown of SRPK1 or inhibition of SRPK1-mediated SRSF1 phosphorylation switches the balance of VEGF splicing to increase the levels of the corresponding anti-mitotic/anti-angiogenic isoform (24, 28), our data indicates that, in GH4C1 cells, SRPK1 inhibition reduces the expression of the pro-survival VEGF164a isoform but other splicing factors may be involved in the usage of distal splicing site in VEGF gene. A third scenario was reported by Gammons and coworkers, where melanoma A375 shRNA SRPK1 tumours showed a slower growth rate than controls and although VEGF165a protein was reduced in knockdown tumours compared with controls anti-angiogenic VEGF165b isoform remained unchanged (39, 46). It is worth mentioning that SRPK1/SRSF1-induced changes in alternative splicing affect several key genes and pathways involved in tumoral transformation (e.g. Ras-MAPK and PI3K-mTOR) (13) or apoptosis regulation (e.g. BIN1, BIM, BCLX (BCL2L1), MCL1, and CASP2 and CASP9), (47, 48), thus other mechanism besides VEGF modulation might be affected in GH4C1 cells. In these cells we also observed an increase in cell apoptosis upon treatment with SRPK1 inhibitors. Since there exist data suggesting that VEGF protects cells from apoptosis (49), we hypothesize that acting on VEGF splicing with SRPIN340 or SPHINX31 could have shifted the balance towards the activation of pro-apoptotic pathways, or/and, as just mentioned, other off-targets mechanisms downstream SRPK1/SRSF1 besides VEGF could have triggered apoptosis pathways.

In addition, SRPIN340 as well as SPHINX31 played a strong inhibitory effect on cell migration in GH4C1 cells. This is particularly of interest since in previous reports a correlation between VEGF expression and suprasellar extension has been observed in a group of aggressive GH-secreting PitNETs (50, 51). Here, to verify the hypothesis that the anti-tumoral effects of SRPIN340 and SPHINX31 were related to a downstream reduction of SRSF1-induced VEGF164a isoform production, we analysed data coming from GH4C1 cells co-stimulated with the recombinant VEGF164a protein and SRPIN340 or SPHINX31. Under these experimental conditions, the reduction of cell migration and cell viability mediated by both SRPK1 inhibitors were impaired, clearly indicating that the VEGF164a isoform is responsible, at least in part, for the pro-survival pathways that sustain tumorigenesis in GH4C1 cells. Accordingly, the overexpression of a human VEGF165a cDNA driven by a VEGF-promoter (thus insensitive to alternative splicing) rescued the tumour growth in SRPK1-silenced prostatic adenocarcinoma cells (42). To the best of our knowledge, no studies have been performed to assess whether VEGF splicing isoforms (VEGF165a/165b) are differentially expressed in invasive versus non-invasive PitNETs. Unfortunately, the low amount of tumor samples did not allow us to perform RNA extraction to detect VEGF splicing isoforms in our cohort of GH-PitNETs. Few reports are present in the literature studying the role of VEGF165b in PitNETs and they mostly attempt to correlate the expression levels of this inhibitory variant of VEGF with the low microvessel density (MVD) often observed in most of PitNETs compared to normal pituitary gland. However, these works showed that only a small proportion of PitNETs overexpress VEGF165b, suggesting the existence of other inhibitory mechanisms of tumor angiogenesis in these tumors (34, 52).

Currently, SRLs that target somatostatin receptors (SSTR) are the pharmacological drugs used as first line therapy to treat patients with GH-PitNET in case of unsuccessful debulk of tumor or recurrence. SRLs ability to inhibit VEGF has been poorly investigated in PitNET primary cultures. In a study with non functioning (NF)-PitNET primary cultures, pasireotide inhibited tumour cell viability by, at least in part, reducing VEGF secretion (53). Here, we observed a reduction of VEGF by octreotide and SRPIN340 in one primary culture from a GH-secreting PitNET. Moreover, SRPIN340 was able to exert mild but significant antisecretory action in all GH-secreting primary cultures tested, resulting effective in reducing GH levels also in one primary culture *in vitro* resistant to treatment with octreotide. This finding suggests that reducing the pro-survival VEGF165a isoform by means of SRPK1 inhibitors may represent an alternative strategy to treat SRL-unresponsive aggressive tumors or, in combination with SRLs, it may help to achieve a stronger efficacy in SRLs weak responsive tumors. In order to test this latter hypothesis, further experiments evaluating the effects of a combined treatment need to be performed. In addition, a more direct mechanism could be hypothesized since the alternative splicing of human *GH-1* gene is regulated by SRSF1 that promotes exon 3 inclusion and favours expression of 22 kDa GH isoform (the most abundant GH form in the pituitary) at the expense of the 17.5-kDa shorter and biological inactive variant (54, 55). However, the low number of cells obtained after GH-PitNETs dispersion did not allow us to assess the expression levels of different GH isoforms in primary cultures, thus future studies will investigate whether SRPIN340 can inhibit SRSF1 activity and promote GH isoform switching.

Overall, although large scale studies are missing, preclinical data indicate that VEGF is a potential therapeutic target in PitNETs. Refractory PitNETs and pituitary carcinomas resistant to conventional treatments have proven benefit from anti-VEGF therapies (37). In this scenario, modulating VEGF alternative splicing by small molecule SRPK1 inhibitors could allow to overcame side effects of bevacizumab and antibody-based therapy (56). It has to be mentioned that in our study we could not find any difference in the potency of the biological responses triggered by SRPIN340 and SPHINX31 in GH4C1 cells. Being SPHINX31 a more potent and systemically active molecule than SRPIN340 (39), further studies will explore its in *in vivo* potential in GH-secreting PitNET xenograft models in mice. Indeed, we are aware that the lack of *in vivo* models represents a limitation of the present work. Another potential limitation of our work is the use of GH4C1 cells instead of GH3 cells, the cell model most commonly used in functional studies on GH-PitNET. However, since we detected very low expression level of SRPK1 in GH3 cells in compared to GH4C1 cells, they failed to represent a suitable model for the study of SRPK1 inhibitor.

In conclusion, the present work shed new light on SRPK1 inhibition as a possible novel pathway for further investigation on alternative strategy to exert anti-tumoral effects in pituitary somatotroph cells.

## Data Availability

The raw data supporting the conclusions of this article will be made available by the authors, without undue reservation.
