# Supplementary figures and images for: Potential antitumoral effects of SRPK1 inhibition through modulation of VEGF splicing in pituitary somatotroph tumoral cells
Source: Front Endocrinol (Lausanne). 2025 Oct 8;16:1667327. doi: 10.3389/fendo.2025.1667327 (PMC12540077; doi:10.3389/fendo.2025.1667327)

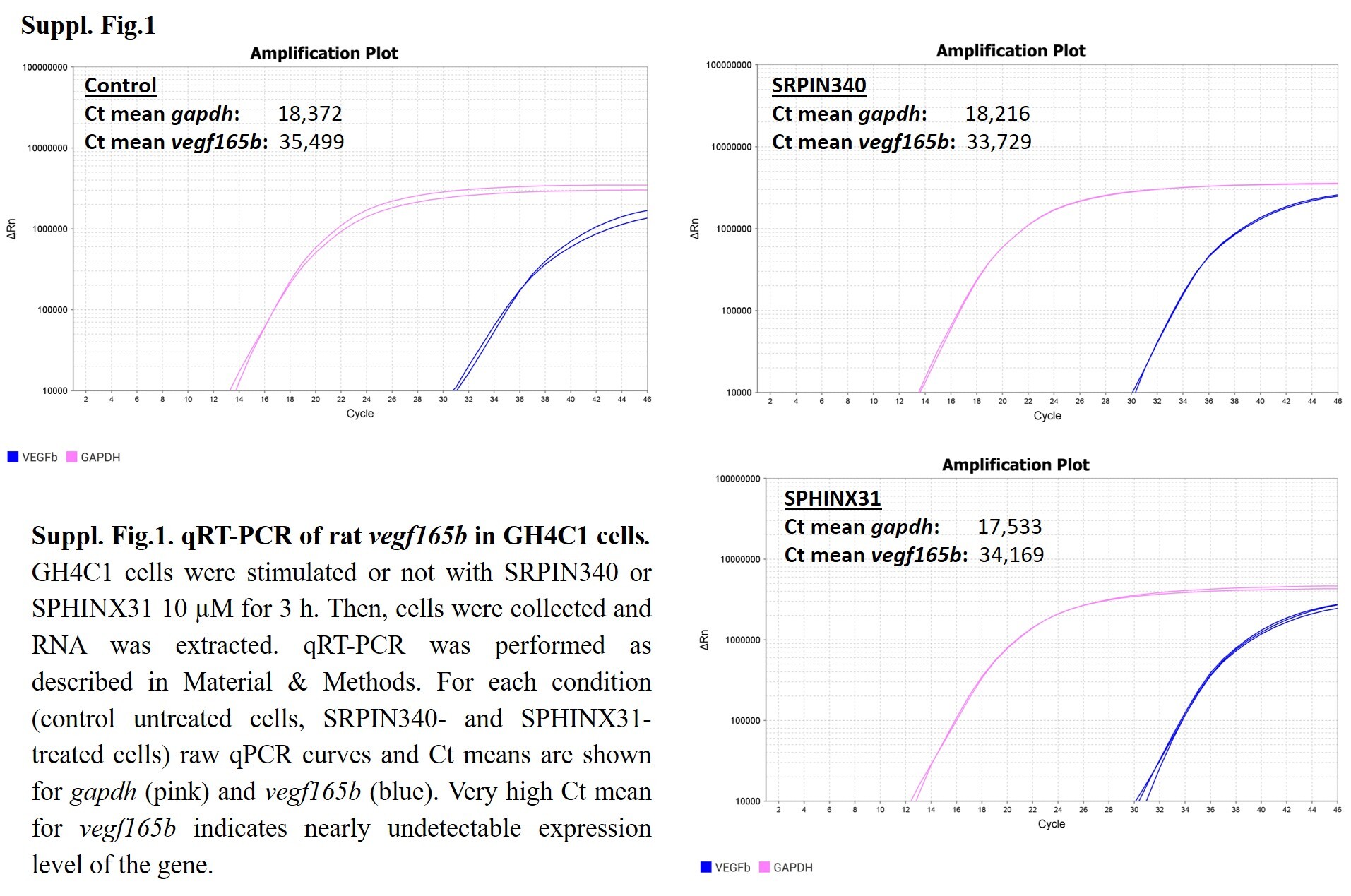

Supplement: Supplementary file 1 [file Image1.jpg]

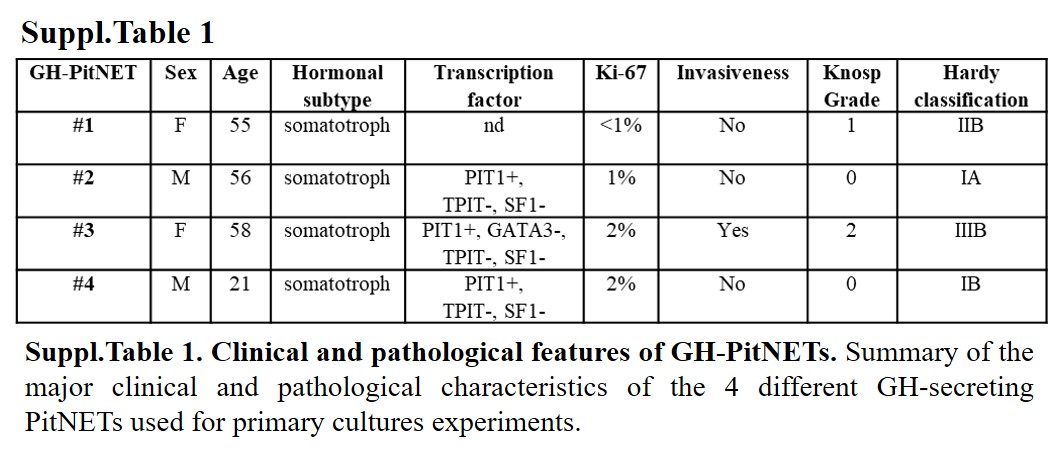

Supplement: Supplementary file 2 [file Supplementaryfile1.jpeg]
